# Development, Modeling, Fabrication, and Characterization of a Magnetic, Micro-Spring-Suspended System for the Safe Electrical Interconnection of Neural Implants

**DOI:** 10.3390/mi9090424

**Published:** 2018-08-23

**Authors:** Katharina Hoch, Frederick Pothof, Felix Becker, Oliver Paul, Patrick Ruther

**Affiliations:** 1 Department of Microsystems Engineering (IMTEK), University of Freiburg, 79110 Freiburg, Germany; katharina.hoch@imtek.uni-freiburg.de (K.H.); frederick.pothof@imtek.uni-freiburg.de (F.P.); felix.becker@imtek.uni-freiburg.de (F.B.); paul@imtek.de (O.P.); 2 BrainLinks-BrainTools Cluster of Excellence, University of Freiburg, 79110 Freiburg, Germany

**Keywords:** neural interfacing, micro-electromechanical systems (MEMS) technologies, microelectromechanical systems, neuroscientific research, magnetic coupling, freely-behaving

## Abstract

The development of innovative tools for neuroscientific research is based on in vivo tests typically applied to small animals. Most often, the interfacing of neural probes relies on commercially available connector systems which are difficult to handle during connection, particularly when freely behaving animals are involved. Furthermore, the connectors often exert high mechanical forces during plugging and unplugging, potentially damaging the fragile bone structure. In order to facilitate connector usage and increase the safety of laboratory animals, we developed a new magnetic connector system circumventing the drawbacks of existing tools. The connector system uses multiple magnet pairs and spring-suspended electrical contact pads realized using micro-electromechanical systems (MEMS) technologies. While the contact pad suspension increases the system tolerance in view of geometrical variations, we achieved a reliable self-alignment of the connector parts at ±50 µm provided by the specifically oriented magnet pairs and without the need of alignment pins. While connection forces are negligible, we can adjust the forces during connector release by modifying the magnet distance. With the connector test structures developed here, we achieved an electrical connection yield of 100%. Based on these findings, we expect that in vivo experiments with freely behaving animals will be facilitated with improved animal safety.

## 1. Introduction

The goal of neuroscientific research is to analyze brain functionality, and to understand and treat neuronal disorders. For this, neuroscientists and physicians use neural implants to record or trigger neural activity on the brain surface as well as directly within the brain tissue. During the past decades, extensive research in the field of neurotechnology targeting the continuous development and improvement of neural implants has helped to establish appropriate diagnostic methods and clinical treatments for a broad variety of neural diseases. Exemplary disorders such as Parkinson’s disease, epilepsy and dystonia, for which drug treatment might become ineffective over time, can nowadays be successfully targeted by deep brain stimulation or diagnosed by neural implants followed by a subsequent surgical resection of the affected brain areas [1,2,3,4], respectively. Furthermore, auditory and visual prostheses such as the well-established cochlear implants or innovative retina implants are based on the ongoing improvement in recording and stimulation techniques [5,6,7,8].

Basic neuroscientific research is most often performed on small animals such as rodents [9]. In the case of translational efforts towards clinical applications, neuroscientific tools and procedures need to be further approved in larger animals such as non-human primates (NHP) [10,11,12], sheep [13] or pigs [14]. Depending on the complexity and duration of these experiments, probes to be implanted and validated are interfaced either by transcutaneous connectors [15] or via fully implantable, wireless recording systems [16].

**Figure 1:**
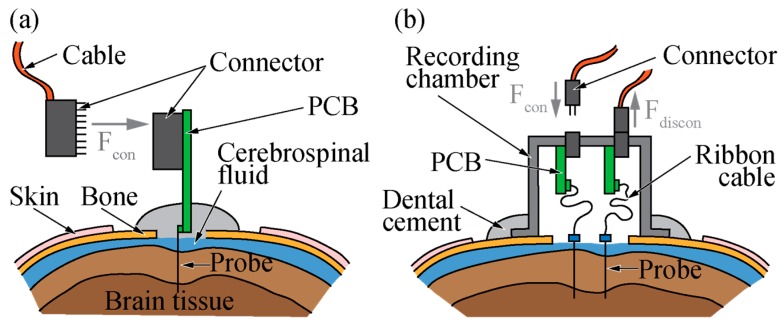
Silicon-based probes (**a**) directly interfacing a printed circuit board (PCB) fixed to the skull using dental cement, and (**b**) floating with the brain interfacing a PCB fixed to a recording chamber. The electrical interface between probe and an external instrumentation is accomplished via small strip connectors providing a tethered connection ($F_{con}$ and $F_{discon}$ represent forces exerted during connection and disconnection, respectively).

Figure 1a illustrates a recording experiment using a silicon-based neural probe implanted into the cortex of a rat. As sketched, the neural probe is attached to a printed circuit board (PCB) carrying a small strip connector, e.g., of the Omnetics Nano Series (Omnetics, Minneapolis, MN, USA) [17,18] or MOLC/FOLC (Samtec, New Albany, IN, USA) type [19]. Following probe implantation, the probe and PCB are permanently anchored to the skull using dental cement, which at the same time seals the craniotomy that exposed the brain surface prior to probe implantation. Obviously, the skulls of mice and rats require careful handling during surgery. In particular, when connecting and disconnecting the external instrumentation either directly or via a longer tether cable (Figure 1a) using the aforementioned connectors, excessive force may easily damage the fragile bone structure.

The example of a recording chamber, as often used in the case of NHP experiments to mechanically protect the surgical area as well as the connectors between the various recording sessions, is shown in Figure 1b. In this case, the neural implant is interfaced to a small connector fixed in the recording chamber via a highly flexible ribbon cable connected to an intermediate PCB [10,20]. When the connector is being plugged in, connecting forces $F_{con}$ are effectively distributed across the chamber sidewalls avoiding local mechanical stress peaks on the skull surface. In contrast, unintended forces pulling the cable in different directions relative to the connector orientation might induce torque on the connector, eventually damaging the cable or the connector itself.

The interconnections formed in the case of these specific animal experiments are often quite rigid and need comparably large connection forces. As an example, Omnetics Nano Series connectors require connection and disconnection forces up to 2 N per contact [21]. Forces exerted during connection and disconnection potentially harm the fragile skull of small animals while unintended forces applied during experimental use might damage the connectors or interfacing cables, or harm the bone structure by large mechanical torque. In addition to issues related to excessive or unintended forces and torque, some connector variants are often not intended for repeated connection and disconnection. Wear may result in unreliable electrical connection. Thus, innovative connector systems circumventing the aforementioned issues are required. They need to be laid out for experiments on small animals such as mice and rats, require minimal connection and disconnection forces, and also enable simple and fast handling in the case of experiments with freely behaving animals. Furthermore, the connector needs to be compatible with a broad variety of different devices such as silicon-based probes [20,22], flexible polymer-based electrode arrays [23,24], and other devices. Even the above-mentioned wireless recording systems may benefit from an enhanced connector system, as any headstage—may it be wireless or not—has to be connected to the neural probe in some way.

One technical solution to reduce connecting/disconnecting forces implies magnetic coupling of the two connector parts. Such a magnetic break-away connection was initially introduced for a deep fryer equipped with a magnetic power cord [25]. This connector variant was spread to a broader audience when Apple applied it in its laptops as the magnetically attached power supply MagSafe^®^ (Apple, Cupertino, CA, USA). The connection is designed such that unintended forces acting on the power cord immediately unclasp the connection, therefore preventing the laptop from being pulled and damaged unintentionally. The connector unit applies a ferromagnetic material and five gold-plated, spring-mounted contacts on one side. The counterpart contains five concave contact pads surrounded by a permanent magnet which provides the force needed to fix both connector parts and at the same time shields the electrical pins. This connector is approximately 17 mm wide and 5 mm tall. *MagSafe* was granted a patent in 2007 [26].

To the best of our knowledge, there are only a few magnetically-coupled connector systems available for neural implants. One example, a magnetic fiber stub for optogenetic stimulation experiments has been developed by Plexon (Dallas, TX, USA) [27]. It is intended to be combined with either a magnetic head-mounted light-emitting diode (LED) or an optical patch cable which contains a magnetic ferrule. The interface comprises a stainless steel tubing and a ring magnet enabling free rotation while being connected to the external component. This effectively reduces the torque on the implant which is highly beneficial for experiments with freely behaving animals.

A percutaneous connector with up to 128 channels using spring-loaded pin contacts has been introduced by Smith and Guillory [28]. The magnetic coupling system comprises a ferrous ring on one connector part and several magnets in the respective counterpart. The precise alignment of both connector halves is achieved by an alignment pin. The magnetic connector system presented by Shah et al. [29] features up to 64 channels. It consists of several magnets circularly mounted around the connector center with alternating magnetization directions of the individual magnets. This design includes an additional anisotropic conductive film (ACF) placed between the two connector halves acting as an interconnect sheet to guarantee a good electrical contact. The alignment of the connector parts is again achieved by means of passive alignment features. Both percutaneous connector variants fulfill the above-mentioned requirements to some extent. Connection and disconnection seem to work without the need of large forces while providing a stable electric transmission. However, the connector dimension renders these devices impractical for experiments with small animals. The alignment pins reduce the ease of operation and prevent disconnection by lateral forces in experiments with freely behaving animals, thus reducing the safety of the animals.

In the present work, we propose a new magnetic connector system offering minimal connection and disconnection forces, a secure disconnection when unintended lateral forces are applied, and fast and easy device handling during connection. The latter advantage is achieved by the omission of alignment pins. Although the connection is less rigid, and thus, the risk of animal harm or device failure is reduced to a minimum, the mechanical system is strong enough to ensure a stable electrical connection. The initial idea of implementing spring-suspended contact pads in a MEMS-based connector and its fabrication were previously presented in [30]. Here we provide a comprehensive description of the novel connector system and the underlying fabrication process, an in-detail mechanical and electrical characterization, as well as an analytical model of the spring-suspended contact pads. The insights gained from this model in conjunction with the experimental system characterization enable the definition of an optimal connector design for future modules to be applied in neuroscientific experiments.

## 2. Materials and Methods

### 2.1. Module Design

**Figure 2:**
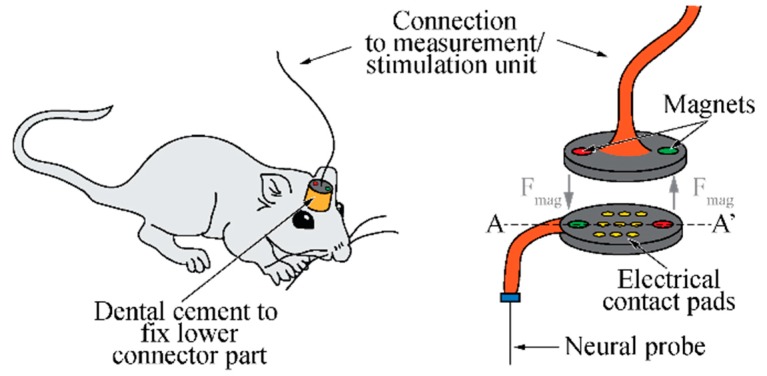
Schematic of basic connector concept applying small magnets to magnetically pull the connector parts together. Different magnet orientations are exploited to self-align the parts when being brought in contact.

The aim of this work is to develop a novel connector system for neural implants. In contrast to most of the existing interconnection technologies, the system is designed to work with low connection and disconnection forces, and thus, to be suitable for experiments with small rodents, among other applications. As illustrated in Figure 2, the connector system comprises two connector parts. They are pulled together by small magnets enabling a reliable signal transmission between adjacent electrical contact pads. While the lower part interfacing the neural probe is permanently fixed to the skull of the animal, the counterpart is equipped with a tether cable giving access to the external instrumentation. As magnets are used, connection forces are negligible while the disconnection force is defined by the strength and number of magnets applied. By installing the magnets in different directions of magnetization, the two connector parts self-align once in close proximity. As no alignment features are needed, the connection is easily released as soon as external forces parallel to the plane of connection exceed a threshold value. This magnetic break-away connection prevents animal harm and device failure.

**Figure 3:**
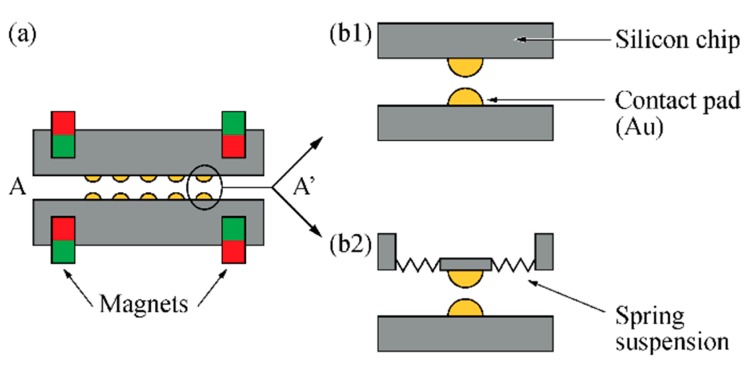
(**a**) Cross-section of connector parts illustrating the adjacent contact pads and (**b**) respective pad compliances using either (**b1**) a stiff pad contact or (**b2**) spring-suspended pads on the upper connector part.

In addition to the magnetic break-away connection, the study compared different contact pad compliances, as illustrated in Figure 3. Protruding gold (Au) bumps realized, e.g., by electroplating were used as electrical contacts. In the case of the stiff connector variant (Figure 3b1), both parts comprise silicon (Si) chips of the same thickness. In contrast to this approach, the connector variant shown in Figure 3b2 applies spring-suspended pads on the upper connector part to account for possible module bow and warp, and for differences in the electroplated pad heights.

**Figure 4:**
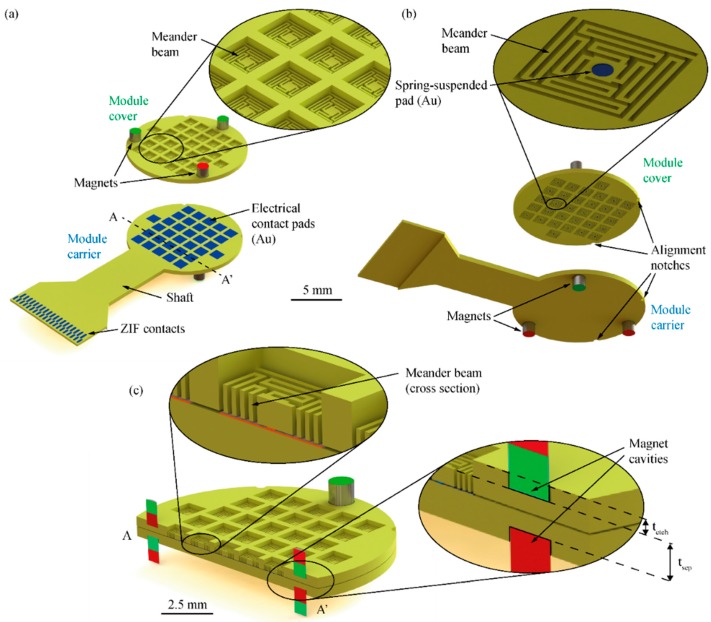
Connector modules seen from (**a**) above and (**b**) below. The module cover comprises spring-suspended pads and three magnets integrated on its top side. The module carrier contains quadratic contact pads connected to a zero insertion force (ZIF) interface and magnets on its bottom side. Cover and carrier comprise additional alignment notches for inspection purposes; magnet orientation is indicated by the red and green color. (**c**) Cross-section of connected modules along line A-A in (**a**). Magnet distance is denoted $t_{sep}$. Adapted from [30].

Connector test modules, as schematically shown in Figure 4, were designed and fabricated in order to investigate the relevant connection forces, contact resistance and interconnection yield. They comprise a module carrier that can be interfaced via zero-insertion force (ZIF) [31] connectors enabling a time-efficient electrical validation of the connector system. A circular module cover (diameter 10 mm, thickness 380 µm) constitutes the upper connector part shown in Figure 2. Depending on the connector variant, as shown in Figure 3b, 32 rigid or spring-suspended contact pads are integrated in the module cover at a center-to-center distance of 1250 µm while the same number of rigid pads are used in the module carrier. In a real connector system used for animal experiments, the module carrier and cover are interfaced to the implantable probe and a tether cable, respectively, as indicated in Figure 2.

The module carrier and cover each comprise three circular cavities (diameter 1 or 1.2 mm, depth ≥ 180 µm) for cylindrical magnets. The cavities are implemented in the module face opposite to the contact pads. As illustrated by the different colors in Figure 4, one of the three magnet pairs is mounted with opposite direction of magnetization. This guarantees that the two halves of the connector can only be mated in a unique orientation.

In order to enhance the compliance of the contact pads and to increase the system tolerance towards deviations in the contact pad heights, as well as the potential bow and warp of the connector modules, one variant of the module cover is equipped with spring-suspended contact pads. The metallized contact pads with a diameter of 150 µm are integrated on quadratic structures with a side length of 250 µm. As shown in Figure 4b, each pad is symmetrically suspended on four meander-shaped beams (total length 2455 µm, width 40 µm, thickness $t_{b}$) within a rigid, quadratic frame (side length 970 µm). The number of meander beam sections and their respective lengths were determined with the help of an analytical model and finite element (FE) simulations. All corners of the meander structure are rounded in order to avoid mechanical stress concentrations. While the rigid frame has a thickness of 380 µm equivalent to the wafer thickness $t_{wafer}$, beams and contact support structures are thinned down to $t_{b}=t_{wafer}-t_{etch}$, where $t_{etch}$ represents the etch depth of the magnet cavities. The beam thickness corresponds to half of the magnet distance $t_{sep}$, as indicated in Figure 4c.

While the rigid contact pads on the module carrier are of quadratic shape (length 930 µm), circular pads (diameter 150 µm) are implemented on the module cover. The relatively large size difference of the pads allows to compensate for in-plane misalignments of both connector parts of up to 840 µm. For test purposes, the electroplated pad heights on the carrier and cover were varied between 1 and 8 µm. Metal lines on the carrier base connecting the pads with the ZIF section along the 4-mm-wide shaft are made of chromium (Cr) and Au.

To facilitate and accelerate the electrical characterization, the test modules were designed in a way that contact pads on the module cover are short-circuited in pairs. In this way, mated connector parts can be efficiently interfaced via the ZIF interface on the module carrier enabling the extraction of connection yield and contact resistance. The lateral alignment of the module cover and carrier relative to each other is validated by optical inspection under a microscope using two semi-circular alignment notches (diameter 500 µm) implemented in the rim of carrier and cover (see Figure 4b).

Cylindrical, nickel-plated neodymium magnets (Supermagnete, Gottmadingen, Germany; height and diameter of 1 ± 0.1 mm) were used for the test modules. They weigh 6 mg and have a maximum energy product of 342–358 kJ/m^3^.

### 2.2. Module Fabrication

**Figure 5:**
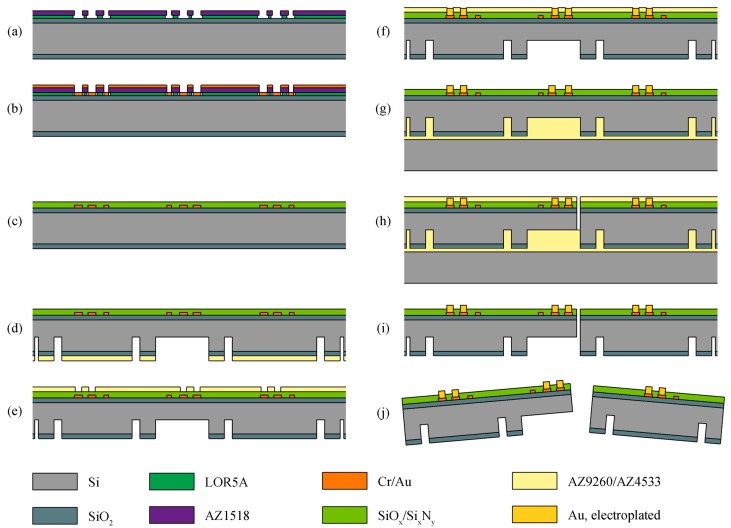
Fabrication process to realize the connector modules by means of microsystems processes performed on the wafer front (**a**–**c**,**h**) and rear (**d**–**g**), followed by the separation of the individual parts (**i**,**j**). Adapted from [30].

The test structures are designed so that module carrier and cover can be fabricated on a common Si substrate using a single process flow. Both module variants presented in Figure 3b were fabricated simultaneously in the same process. Figure 5 summarizes the main clean room fabrication steps for these Si test structure modules. As substrates, 380-µm-thick Si wafers covered by a 1-µm-thick thermal silicon oxide (SiO_2_) layer on both sides were used.

Following the initial cleaning of the wafers in acetone, isopropanol and Piranha solution, a lift-off process was used to pattern the metallization. This applies hexamethyldisilazane (HMDS) as an adhesion promoter, and spin-coating and hard-baking of the 700-nm-thick photo-insensitive resist LOR5A (MicroChem Corp., Westborough, MA, USA) and the 1.8-µm-thick positive photoresist AZ1518 (Microchemicals GmbH, Ulm, Germany). During ultra-violet (UV) light exposure, only the AZ1518 resist is modified, which results in a locally increased solubility. During the subsequent development using tetramethylammonium hydroxide (TMAH), the AZ1518 is dissolved in areas exposed to UV light. In contrast, LOR5A is dissolved isotropically where the structure of the AZ resist gives access to the TMAH solution. Due to this isotropic behavior and a strictly time-controlled development, an undercut of about 1 µm was obtained (see Figure 5a), which facilitated the subsequent lift-off of the metal layer. The metallization, i.e., 50 nm chromium (Cr) and 200 nm Au were sputter-deposited (Figure 5b) with the Cr layer used as an adhesion promoter between the SiO_2_ and Au layers. The resist strip lifts the metal on top of the patterned AZ1518, resulting in well-defined contact pads and conducting leads on the Si substrate.

Patterning the metallization was followed by the plasma enhanced chemical vapor deposition (PECVD) of a 1-µm-thin passivation layer (Figure 5c). Similar to an established fabrication process of Si-based neural probes [32], the passivation comprises a stack of multiple silicon oxide (SiO*_x_*) and silicon nitride (Si*_x_*N*_y_*) layers providing an optimal stress compensation. This passivation also serves as a protection layer for the metal structures during the subsequent process steps carried out on the wafer rear, which brings the wafer front into contact to wafer chucks.

The photolithography on the wafer rear applied another dehydration bake and the adhesion promoter HMDS. Next, a 10-µm-thick layer of the positive photoresist AZ9260 (Microchemicals GmbH) was spin-coated and structured using UV lithography. This serves as the etch mask for the rear SiO_2_ layer patterned using reactive ion etching (RIE). This was followed by deep reactive ion etching (DRIE) of Si (see Figure 5d) to define the magnet cavities, the areas under the ZIF pads on the carrier modules, and the areas where the spring-suspended contact pads will be realized in the module covers. To obtain test structures with different suspension beam thicknesses, the etch depth was varied between 200 µm and 330 µm. In addition, the outer rear shape of the individual components was defined in this step.

Stripping the photoresist in acetone and isopropanol, the wafer processing continued with a further photolithography step on the front to open the passivation at the contact pads that will be thickened by electroplating. Again, a 10-µm-thick layer of AZ9260 was spin-coated and structured by UV lithography (Figure 5e) followed by RIE to open the passivation. Subsequently, the contact pads were electroplated at a current density of 0.75 mA/cm^2^ resulting in a deposition rate of approximately 0.0475 µm/min according to Faraday’s law of electrolysis. Using the AZ9260 resist as a masking layer, wafers of different contact pad heights were realized. Following electroplating, the AZ9260 resist was stripped and the wafer was transferred onto a handle wafer fixed using the resist AZ4533 (Microchemicals GmbH) (see Figure 5g). The handle wafer is needed for stability reasons, especially for the final front etching, where the Si parts are separated from each other.

The next photolithography step was carried out on the wafer front, again using a dehydration bake, HMDS-based adhesion promotion as well as spin-coating and patterning of a 10-µm-thick layer of AZ9260. The resist was used as a mask in the RIE process to open the passivation layer. This was followed by a wet etch to remove the electroplating feed lines of the individual connector parts. The Au was etched with a potassium iodide (KI)-iodine(I_2_)-solution (100 g KI and 25 g I_2_ plus deionized (DI) water for 1 L of etch solution), whereas the Cr was removed with a ceric ammonium nitride ((NH4)_2_Ce(NO_3_)_6_)-nitric acid (HNO_3_) solution (165 g (NH_4_)_2_Ce(NO_3_)_6_ and 90 mL HNO_3_(69%) plus DI water for 1 L of etch solution). By means of DRIE, the wafer was then etched through to a depth ≤ 180 µm to achieve the trenches defining the geometry of the individual parts, which have already been pre-etched from the wafer rear (Figure 5h). The struts used to suspend the connector parts in the fabrication wafer were not etched from the wafer front.

After a final resist strip and release of the handle wafer, the Si components were suspended inside the fabrication wafer by the struts (Figure 5i). Individual parts were separated by applying torsional forces with the aid of a pair of tweezers (Figure 5j). This was then followed by the assembly and the fixation of the magnets in their cavities.

### 2.3. Modeling of Spring-Suspended Contact Pads

#### 2.3.1. Analytical Model

**Figure 6:**
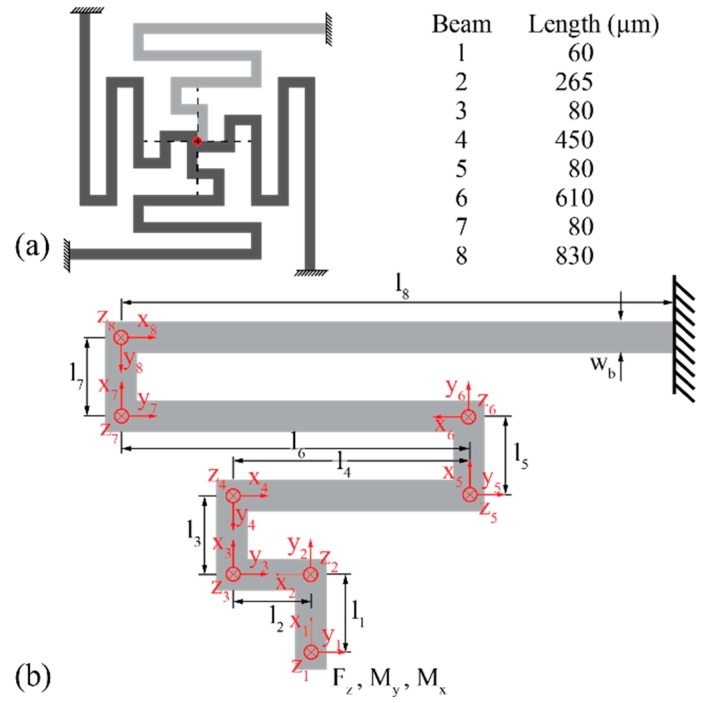
(**a**) Symmetric suspension beam system comprised of four identical meander beams fixed to the rigid frame and lengths of the individual beam sections. (**b**) Mechanical model of one meander beam consisting of eight beams sections (lengths $l_{i}$ (i=1, …, 8)) of width $w_{b}$ and thickness $t_{b}$. The model applies the Young’s modulus $E_{Si}$, area moment of inertia ***I***, shear modulus $G_{Si}$ and torsional moment of inertia $I_{t}$. The force $F_{pad}$ applied to the complete suspension system is translated into the force $F_{z}$ = 0.25 $F_{pad}$ and the moments $M_{x}$ and $M_{y}$ acting on a single meander beam ($F_{z}$, $M_{x}$ and $M_{y}$ are defined in the global ***x-y-z***-coordinate system).

An analytical model was derived to describe the mechanical behavior of the meander-shaped contact pad suspension. It considers both bending and torsion of the individual sections of the suspension beam and is based on the schematic shown in Figure 6a. As the suspension system with its four identical suspension beams is symmetric, only one of the beams is modeled, as illustrated in Figure 6b.

Each of the meander-shaped beams has one fixed support where the eighth beam section is connected to the Si frame which is assumed to be perfectly rigid. The other end is connected to the quadratic contact pad support onto which the force $F_{pad}$ acts in the positive z-direction when the connector modules are brought into contact. Each beam section *i* (i=1, …, 8) is described by its respective length $l_{i}$, the area moment of inertia $I=w_{b}t_{b}^{3}/12$ where $w_{b}$ and $t_{b}$ denote the beam width and height, respectively, the torsional moment of inertia $I_{t}=t_{b}w_{b}^{3}/3\left(1-0.630w_{b}/t_{b}+0.052w_{b}^{5}/t_{b}^{5}\right)$ [33], as well as the Young’s modulus $E_{Si}$, Poisson’s ratio $\nu_{Si}$, and the shear modulus $G_{Si}=E_{Si}/2\left(1+\nu_{Si}\right)$ of Si. Local coordinate systems $x_{i}$-$y_{i}$-$z_{i}$ (i=1, …, 8) are used in each beam section *i* to define local internal forces and moments.

As indicated in Figure 6a, the pad suspension comprises four identical meander-shaped beams connected to a quadratic structure supporting the contact pad. In order to analyze the mechanical behavior of this beam system when a vertical force $F_{pad}$ is applied in its center (see red dot in Figure 6a), the model evaluates one quarter only, obtained by cutting the pad suspension along the dashed lines shown in Figure 6a. The external force $F_{pad}$ acting on the entire suspension is translated in forces $F_{j}$ and moments $M_{j}$ with j=x, y, z acting on each individual meander beam. For symmetry reasons and because beam deflections were kept small, the model assumes that $F_{x}\;=\;F_{y}\;=\;0$ and $M_{z}\;=\;0$. This leaves the two moments $M_{x}$ and $M_{y}$, shown in Figure 6b, as unknowns while the force $F_{z}$ in z-direction equals $F_{pad}/4$. The moments together with the mating force $F_{z}$ can now be transferred through the meandering suspension beam to calculate the internal forces and moments along the influence line resulting in the bending and torsional moments $M_{y_{i}}$ and $M_{x_{i}}$ as functions of $x_{i}$ (i=1, …, 8), respectively. They are listed in Table 1.

Equation (1) in Table 1 can be summarized for *k* > 0 to

(1) $$\;M_{y_{k}}\left(x_{k}\right)=-F_{z}x_{k}-{\left(-1\right)}^{\frac{k\left(k+1\right)}{2}}M_{x_{k-1}}$$

(2) $$\;M_{x_{k}}\left(x_{k}\right)={\left(-1\right)}^{\frac{k\left(k+1\right)}{2}}M_{y_{k-1}}\left(l_{k-1}\right)$$

with $M_{y_{0}}\left(l_{0}\right)=M_{x}$ and $M_{x_{0}}=-M_{y}$ as used in Table 1. The term ${\left(-1\right)}^{k\left(k+1\right)/2}$ generates the sequence (−1, −1, 1, 1, −1, −1, 1, 1, …) for all natural numbers k>0.

In order to calculate the deflection of the pad suspension shown in Figure 6, the analytical model applies the energy theorem of mechanics that states that the work W done by external loads on a deformed body equals the stored elastic energy ∏ [34]. All further formulas in this section are taken from [35]. Consequently, the work carried out on the beam system by applying a force $F_{pad}$ during connector mating is entirely transformed into elastic energy.

The specific strain energies $\prod_{b,i}^{*}$ and $\prod_{t,i}^{*}$ of bent and twisted beams, respectively, are given by

(3) $$\prod_{b,i}^{*}=\frac{1}{2}\frac{M_{y_{i}\;}^{2}}{E_{\mathrm{Si}}I}$$

(4) $$\prod_{t,i}^{*}=\frac{1}{2}\frac{M_{x_{i}}^{2}}{G_{\mathrm{Si}}I_{t}}$$

where $M_{y_{i}}$ and $M_{x_{i}}$ denote the bending and torsional moments acting on the *i*-th beam section, as given in Equation (1). The specific strain energies, which are given per unit length, are integrated over the total beam length $l_{b}=\underset{i}{\sum }l_{i}$ to achieve the total strain energy $\prod_{\mathrm{tot}}$ of the spring-suspension given by

(5) $$\prod_{\mathrm{tot}}=\int_{0}^{l_{b}}\left(\prod_{b}^{*}+\prod_{t}^{*}\right)dx$$

With the bending and torsional moments from Equation (1), one separately calculates the respective contributions of the individual beam sections *i* to the bending and torsional energies $\prod_{b,i}$ and $\prod_{t,i}$. They are listed in Table 2, normalized by ${\left(2E_{\mathrm{Si}}I\right)}^{-1}$ and ${\left(2G_{\mathrm{Si}}I_{t}\right)}^{-1}$, respectively.

Summarizing over all beam sections, the total strain energy $\prod_{\mathrm{tot}}$ is given by

(6) $$\prod_{\mathrm{tot}}=\frac{1}{E_{\mathrm{Si}}I}\left(A_{1}F_{z}^{2}+A_{2}F_{z}M_{y}+A_{3}M_{y}^{2}+A_{4}F_{z}M_{x}+A_{5}M_{x}^{2}\right)+\frac{1}{G_{\mathrm{Si}}I_{t}}\left(B_{1}F_{z}^{2}+B_{2}F_{z}M_{y}+B_{3}M_{y}^{2}+B_{4}F_{z}M_{x}+B_{5}M_{x}^{2}\right)$$

with $A_{m}$ and $B_{m}$ being functions of the beam lengths $l_{i}$ (*i* = 1, …, 8), i.e., $A_{m}=f_{m}\left(l_{1},\ldots ,\;l_{8}\right)$ and $B_{m}=g_{m}\left(l_{1},\ldots ,\;l_{8}\right)$ for m=1, …, 5.

The result is an expression of the total strain energy $\prod_{\mathrm{tot}}$ with three unknowns acting on the central pad support, i.e., the force $F_{z}$, the bending moment $M_{y}$ and the torsional moment $M_{x}$.

Castigliano’s first theorem calculates the displacement or the angular rotation at the point of application of a force and moment in the direction of the force line of the causative force and the rotation axis of the causative moment, respectively. For this purpose, the derivatives of the total strain energy $\prod_{\mathrm{tot}}\left(F_{i},\;M_{i}\right)$ as a function of the applied external forces and moments are taken with respect to the relevant forces or moments. Here these are the vertical displacement,

(7) $${∆}_{z}=\frac{\partial \prod_{\mathrm{tot}}}{\partial F_{z}}$$

and the two rotation angles $\varphi_{n}$, with n=x, y, given by

(8) $$\varphi_{n}=\frac{\partial \prod_{\mathrm{tot}}}{\partial M_{n}}$$

Due to symmetry considerations at the contact pad support, torsion around the $x_{1}$- and $y_{1}$-axis is zero at the transition from the meander beam to the contact pad. Castigliano’s first theorem can therefore be used to calculate $M_{y}$ and $M_{x}$ as a function of $F_{z}$ to fulfill this request. In other terms one has to solve

(9) $$\frac{\partial \prod_{\mathrm{tot}}\left(F_{z},M_{y},M_{x}\right)}{\partial M_{y}}=0$$

and

(10) $$\frac{\partial \prod_{\mathrm{tot}}\left(F_{z},M_{y},M_{x}\right)}{\partial M_{x}}=0$$

It can be shown that $M_{x}$ and $M_{y}$ are linear functions of $F_{z}$ through the origin, i.e., $M_{x}=C_{1}F_{z}$ and $M_{y}=C_{2}F_{z}$ with $C_{p}=h_{p}\left(l_{1},\ldots ,\;l_{8}\right)$ for p=1, 2.

As a consequence, one obtains an expression for the total strain energy $\prod_{\mathrm{tot}}$ which only depends on $F_{z}^{2}$. By taking the derivative of this expression with respect to $F_{z}$, one obtains the displacement of the contact pad support in the z-direction given by

(11) $$∆z=\frac{\partial \prod_{\mathrm{tot}}}{\partial F_{z}}$$

Following Equations (6) and (11) with a quadratic dependence of $\prod_{\mathrm{tot}}$ on $F_{z}$, one obtains a linear relation between ∆z and the applied force $F_{z}$.

#### 2.3.2. Finite Element Simulations

To validate the analytic results and for a fast estimation of the influence of certain parameters, a FE analysis was carried out using COMSOL (Version 5.2a, Burlington, MA, USA). The spring-suspension of an individual contact pad with four symmetrically positioned meander beams was simulated. With a beam width of $w_{b}$ = 40 µm, a beam thickness of $t_{b}$ = 200 µm, and the lengths of the individual beam sections as specified in Figure 6a, the geometrical dimensions were chosen in accordance with the fabricated test structures. As boundary conditions, fixed ends of the meander beams were assumed at the rigid Si frame. A vertical force of $F_{pad}$ = 200 mN corresponding to the total strength of three magnet pairs was applied to the center of the contact pad. The whole structure was defined to be made of Si with a Young’s modulus of 170 GPa and a Poisson’s ratio of 0.28. Passivation layers and metal lines were neglected. The FE simulation was carried out with a free tetrahedral mesh with minimum and maximum element sizes of 0.1 and 10 µm, respectively, a maximum element growth rate of 1.3, a curvature factor of 0.2, and a resolution of narrow regions of 1.

### 2.4. Module Characterization

#### 2.4.1. Mechanical Tests

**Figure 7:**
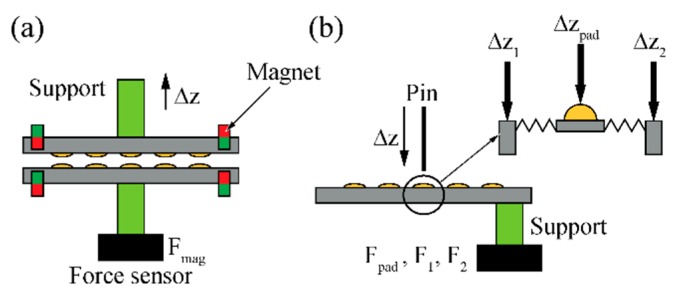
Schematic representation of mechanical testing using a tensile test device; characterization of (**a**) magnetic disconnection force $F_{mag}$ and (**b**) mechanical yield of beam suspension.

The mechanical force $F_{mag}$, which is needed to overcome the attractive force of the magnets and to separate the two connector halves was measured using a tensile test setup (Z2.5 zwicki-Line, Zwick/Roell, Ulm, Germany). In order to fix the connector module components in the test setup using pneumatic clamps, plastic cylinder supports were glued onto the rear sides of individual parts comprising three magnets each (Figure 7a). The magnetic force $F_{mag}$ was recorded as a function of displacement ∆z while separating the two connector parts in the vertical direction at a speed of 0.1 mm/min.

In order to characterize the mechanical behavior and strength of the spring-suspended contact pads, the same tensile test setup was used as well. In this case, we mounted the plastic cylinders used for device clamping off-center on the connector modules comprising the spring-suspensions. With this arrangement we prevented any adhesive entering the trenches between the suspension beams during the assembly process. While the connector module is mounted on the lower clamp, a metal pin (500 µm diameter with sharp tip) is fixed to the movable clamp. The positioning of the pin with respect to the spring suspension is optically controlled using a video microscope. By moving this pin vertically onto the pad, the displacement-force curve $F_{pad}\left(∆z_{pad}\right)$ is recorded. It serves to extract the range of forces that can act perpendicularly on the connector plane without breaking the meander beams. As the fixation pin is glued off-center, we compensated for the compliance of the experimental setup by recording displacement-force curves $F_{q}\left(∆z_{q}\right)$ with q=1, 2 when loading the connector module at two positions on the Si substrate next to the suspension frame, as indicated in Figure 7b. Beam suspension and frame compliance were tested at a speed of 0.1 mm/min of the pin for different meander beam thicknesses.

#### 2.4.2. Electrical Tests

The electrical connection yield was determined by a two-wire resistance measurement. As described in Section 2.1, the contacts on the module cover are short-circuited in pairs. Hence, a two-wire resistance measurement on the ZIF section of the module carrier can determine whether two pairs of Au pads have electrical contact. Should any one of them fail, the measurement will reveal an open loop. A measured resistance below 15 kΩ was rated as a stable electrical connection. This comparably high resistance is explained by the only 6-µm-wide electrical leads between the ZIF section and the contact pads. Different combinations of contact pad heights on the module carrier and module cover were tested.

In order to characterize the contact resistance between two pads, we used three corresponding pads P1 to P3 on the module carrier and cover each. While the three pads on the cover are short circuited with pad P2 being positioned in the middle, pads P1 and P3 on the carrier are interfaced to ZIF pads Z1 and Z4, respectively. In contrast, pad P2 of the module carrier is connected to ZIF pads Z2 and Z3. This enables a four-contact resistance measurement at the mating pads P2 using a wafer prober. A constant current $I_{1,3}$ was applied between the ZIF pads Z1 and Z3 while the voltage drop $∆V_{2,4}$ across the corresponding pads P2 on the module carrier and cover was determined via the ZIF pads Z2 and Z4. This allows the calculation of the contact resistance omitting the line resistances on both connector parts.

## 3. Results

### 3.1. Module Fabrication

**Figure 8:**
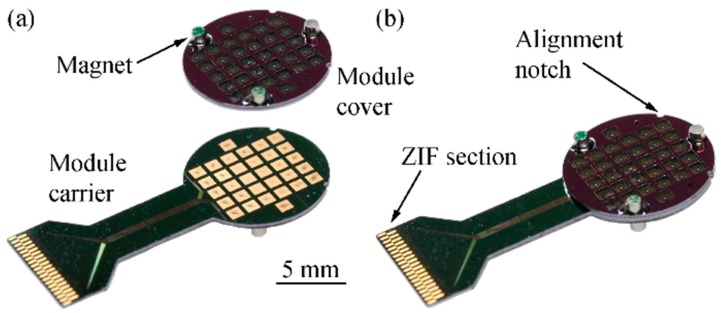
Fully assembled 32-channel Si test structure in the (**a**) separated and (**b**) connected state. Adapted from [30].

**Figure 9:**
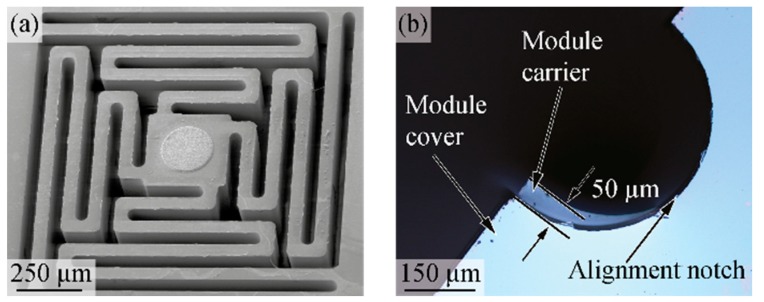
(**a**) Scanning electron micrograph of a spring-suspended contact pad. (**b**) Optical inspection of the self-alignment accuracy of two connector parts using respective alignment notches.

The test structures for the novel connector system were successfully fabricated, as shown in Figure 8. The scanning electron micrograph in Figure 9a shows in detail the spring suspension of a contact pad. By varying the etch depth during rear DRIE, we obtained test modules with meander beam thicknesses of 200 µm, 100 µm, and 50 µm. Consequently, the vertical distance $t_{sep}$ between two magnets in the mated connector state (Figure 4c) was set to 400 µm, 200 µm, and 100 µm.

Using the notches implemented in the rim of the connector parts, the alignment accuracy was optically inspected during repeated connector mating. Following the system idea of magnetic self-alignment described in Section 2.1., no particular care was taken for a precise pre-alignment of the connector halves during the manual connection procedure. The optical inspection of mated connectors revealed a lateral alignment accuracy of better than 50 µm for each of the individual tests (cf. Figure 9b). It has to be kept in mind that the misalignment originates from a combination of relative translation and rotation of the two connector halves. As the misalignment is measured at the connector rim, the observed value represents the worst case, in particular for a pure rotational misalignment.

### 3.2. Modeling

The analytical model demonstrated that the displacement ∆z of a spring-suspended contact pad depends linearly on the mating force $F_{pad}$. With the geometrical beam dimensions given in Figure 6a and the material properties of Si, i.e., $E_{Si}$ = 170 GPa, $\nu_{Si}$ = 0.28 and $G_{Si}$ = 66.4 GPa, the spring constant $c_{model}$ of the meander beam system is extracted on the basis of Equations (6) and (11) as

(12) $$\;c_{model\;}=4\frac{F_{z}}{∆z\left(F_{z}\right)}=31.5\frac{\mathrm{mN}}{\text{µ}m}\;$$

Using realistic magnetic forces of the three magnet pairs of 264 mN and the fact that 32 contact pads are implemented, the force $F_{pad}$ per contact is 8.25 mN equivalent to a displacement of only ∆z = 0.26 µm. This displacement might not be sufficient to overcome device bow, warp, and potential differences in the electroplating pad height, to form a stable electrical connection. To compensate for a certain under-dimensioning of the magnets used in this study we added an additional force of 460 mN by means of an additional weight placed on top of a connector cover. This results in a force of approximately 23 mN per contact and a theoretical pad displacement of 0.72 µm using the modelled spring constant.

**Figure 10:**
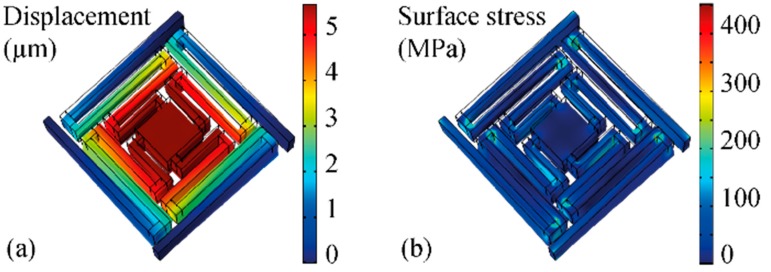
COMSOL FEA of a spring-suspended contact pad (vertical load 200 mN). (**a**) Displacement of the meander beam system. (**b**) Von Mises surface stress due to bending and torsion.

Figure 10 shows the results of the COMSOL finite element simulations for a contact force of 200 mN acting on a single spring-suspended pad. As indicated in Figure 10a, a vertical displacement of 5.67 µm is obtained. This corresponds to a spring constant of $c_{sim}$ = 35 mN/µm, slightly higher than $c_{model}$ above. For this contact force, a maximum von Mises stress of 339 MPa is obtained in the surface of the beam suspension using the FE simulations (Figure 10b). This stress level is below the mechanical yield strength of Si reported to be in the range of 1 GPa [36] up to 7 GPa [37], depending on surface quality. Taking into account that the force used in the FE simulations is well above the applicable force using the chosen magnets, we expect that the mechanical strength of the suspension beams is more than sufficient.

### 3.3. Module Characterization

**Figure 11:**
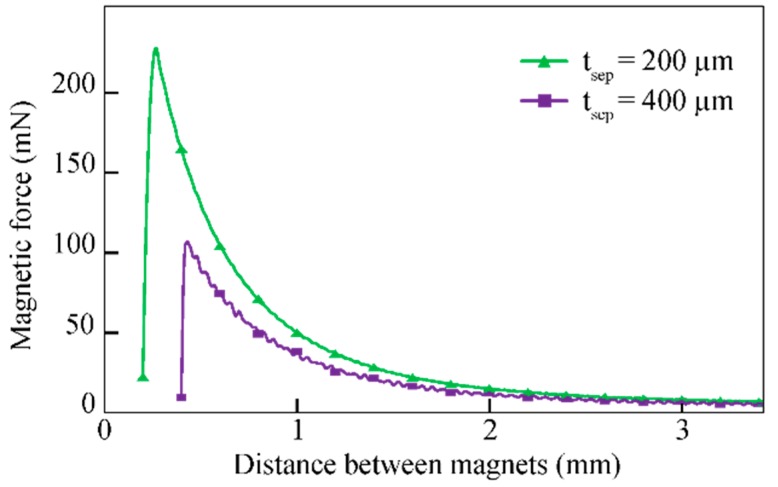
Magnetic disconnection force for two values of $t_{sep}$ as a function of the vertical distance between the magnets. Adapted from [30].

Figure 11 shows the vertical disconnection force $F_{mag}$ of the realized test structures as a function of the vertical displacement ∆z for different magnet distances $t_{sep}$. For $t_{sep}$ = 200 µm and 400 µm, maximum forces of 227 mN and 106 mN have been extracted, respectively. The fact that the two curves do not match exactly is explained by an unknown amount of epoxy inside the magnet cavities increasing the distance between the two magnets in the mated connector state.

**Figure 12:**
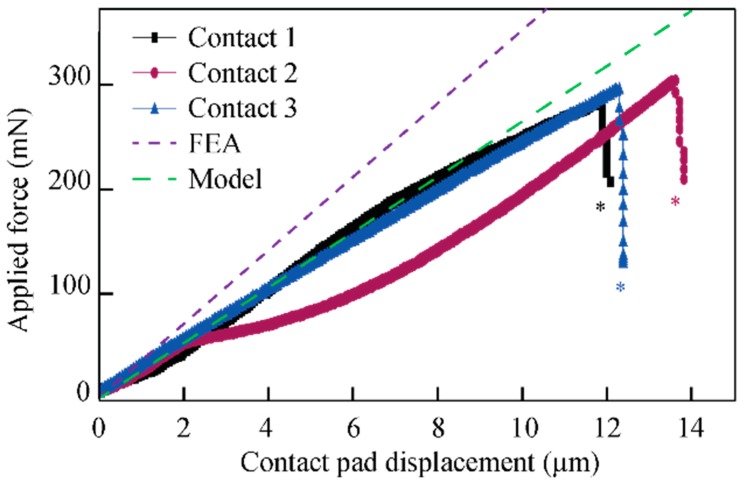
Vertical deflection of suspended contact pads vs applied force comparing experiment (three different pads), analytical model and FE simulations. Beam fracture is indicated by asterisks. Adapted from [30].

Figure 12 shows the deflection of three representative spring-suspended contact pads under a vertical load $F_{pad}$ (symbols). It is compared to the modeled and simulated data, indicated by the solid and dashed lines, respectively. The meander structures typically collapsed at vertical deflections between 11.9 µm and 13.6 µm and a respective force in the range of 300 mN. Following the FE simulations, the maximum force can be translated into a maximum von Mises stress in the beam structure of ca. 500 MPa. The fact that this value is below the data in the literature data [36,37] can be explained by the increased surface roughness of the DRIE processed beam sidewalls.

The electrical characterization of connector modules with different contact heights $h_{cover}$ and $h_{carrier}$ on the module cover and carrier, respectively, is summarized in Table 3. It provides the connection yield as the percentage of successful electrical interconnection of 16 pairs of dual pad connections for the cover modules with spring-suspended (bold) and stiff contact pads (Figure 3b). It should be noted that the actual yield might be higher than the given numbers as a successful contact requests two pads to be effectively interfaced (see Section 2.1) In addition to the connection force exerted by the integrated magnets, an additional force of approximately 460 mN had to be applied to achieve a stable connection in the case of the spring-suspended contact pads. Depending on the pad height combination and thickness of the meander beam, an electrical connection yield of up to 100% was achieved for the spring-suspended variant. In the case of the stiff contact pads, despite the additional force weight applied, we could not achieve adequate percentages of stable electrical contacts.

Each connector configuration, i.e., combination of pad heights $h_{cover}$ and $h_{carrier}$ and beam thickness $t_{b}$, was tested with one module comprising 32 channels, equivalent to 16 pairs of dual pad connections. As the statistical significance of our data is low, only certain trends in view of promising pad height combinations can be extracted. The contact resistance extracted using the 4-point-measurements, as described above, is in the range of 11 to 20 mΩ.

## 4. Discussion

The analytical model and the FE simulation provided slightly different spring constants $c_{model}$ and $c_{sim}$ (see also curves in Figure 12) of 31.5 and 35 mN/µm, respectively. A reason for this difference is certainly that the area moment of inertia used in the analytical model is assumed to be constant along the entire meander length. However, the beam corners have a considerably larger area moment of inertia than the beams. This likely explains the larger stiffness extracted from the FE simulation compared to the analytical model.

The discrepancy between FE simulations and the experimental tests of the beam suspension (Figure 12) can be explained by geometrical variations in the test structure dimensions due to the DRIE fabrication process, e.g., slightly tapered sidewalls, and the fact that the passivation layer and metal leads have not been included in the FE model. In the current design, the pad suspensions are somewhat stiff and this will be addressed in a future design optimization, where modified design parameters as well as alternative suspension geometries will be considered. Such alternative design may be based on helical designs with a reduced number of beams, ultimately enabling a size reduction with potentially smaller pad distances.

In view of system applicability, one has to consider the mechanical strength of the beam suspension, the forces exerted by the integrated magnets, the resulting connection yield and the alignment accuracy of both connector parts. For the given system dimensions, we extracted maximum vertical pad deflections between 11.9 and 13.6 µm at forces of ca. 300 mN per contact pad, i.e., a total force of 9.6 N for an entire connector comprising 32 pads. In that sense, the total pad height $h_{1}+h_{2}$ of the module cover and carrier pads has to be limited to approximately 12 µm assuming that the magnetic force per pad can reach values of 300 mN. However, implementing the given small cylindrical magnets and a separation distance of 400 µm between the magnets, a maximum connection force of 264 mN for 32 pads, i.e., 8.25 mN per pad, cause beam deflections of only 0.26 µm. These deflections are well below the experimentally determined yield strength. Assuming a separation distance of $t_{sep}$ = 0 µm achievable for $t_{etch}=t_{wafer}$, the connection force is increased to 1029 mN, still far below the maximum applicable deflections. Consequently, stronger, yet similarly compact magnets are required and have to be chosen such that the system dimensions are not increased too much.

The higher electrical connection yield of the spring-suspended connector variant in comparison to the stiff version successfully demonstrated the chosen connector concept. A connection yield of 100% for certain pad height combinations was, however, only achievable by applying an additional load of 460 mN on the entire connector. This clearly indicates that either more or stronger magnets need to be implemented in a redesign of the connector. The data summarized in Table 3 indicates that thicker pads on the module carrier, i.e., up to 8 µm, are not suitable for a stable electrical connection between the connector halves. Instead, using slightly protruding pad metallization on the cover module with $h_{cover}$ = 4 µm provides the best results for $h_{carrier}$ between 1 and 4 µm. As discussed in Section 3.3, these findings need to be further validated by a statistically more significant number of test devices.

The lateral self-alignment accuracy of 50 µm is far better than needed for the applied pad sizes of 970 × 970 µm^2^. A pronounced reduction in the pad size is thus possible. However, the current bottleneck in size reduction is the area occupied by the pad suspension relying on the proposed meander beam design.

The disconnection forces, in the range of several hundred mN per connector, are much smaller than those reported for other state-of-the-art magnetic connector systems. As an example, Shah et al. reported a normal disconnection force of 4.9 N for their connector system [29]. Hence, the implementation of stronger magnets as indicated by the applied models and the connection yield tests is still feasible without affecting animal safety.

## 5. Conclusions

A novel connector system for the safe electrical interconnection of neural implants based on a magnetic break-away mechanism was designed, fabricated, and extensively characterized. The system comprises spring-suspended contact pads compensating for the potential bow and warp of the connector components and differences on electroplated pad heights. The disconnection forces of the connector system are clearly reduced compared to other state-of-the-art devices. Therefore, the proposed design offers increased safety for future in vivo applications with freely moving animals. In addition, excessive lateral forces are inherently prevented, thus avoiding the action of mechanical torque on the connectors. Such undesired mechanical loads potentially harm the bone structure of the animal to which the connector is fixed. Safety is further increased by the omission of alignment pins, which is a unique feature of the new connector system.

The high self-alignment accuracy of better than 50 µm during connector mating clearly facilitates system handling when preparing the animals for a recording session. The correct positioning of the connector parts is inherently achieved by the specific arrangement of three magnet pairs. No manual pressure or any mechanical alignment features are needed. Compared to compact connectors as typically used in neuroscientific research, the connection procedure is straightforward and requires no specific training of the end-user.

Our study successfully demonstrated that the spring-suspension provides a high connection yield compared to a stiff connector variant. We identified pad height combinations on the two connector halves that showed the best performance regarding height compliance and thus electrical connection yield. As the high yield was only achieved by applying additional load exceeding the mating forces provided by the integrated magnets, future design optimizations of the connector need to implement more or stronger magnets.

So far, our magnetic, micro-spring-suspended system meets every demand of neuroscientific in-vivo experiments. Still, this has to be verified under real conditions.

The experimental findings regarding the mechanical suspension beam behavior were supported by a simplified analytical model derived in this study to describe the meander-like beams and by finite element simulations. Both the model and simulation predict similar spring constants. This knowledge will be used to define an optimal, application-specific design and respective fabrication parameters for future connector modules directly applicable in neuroscientific research.

## Figures and Tables

**Table 1:** Bending and torsional moments $M_{y_{i}}$ and $M_{x_{i}}$, respectively, of the individual beam sections ***i*** (i=1, …, 8) used in the analytical model shown in Figure 6 assuming that the force $F_{z}$ and the moments $M_{x}$ and $M_{y}$ are applied on a single, meander-shaped suspension beam.

*i*	$\;M_{y_{i}\;}\left(x_{i}\right)$	$\;M_{x_{i}\;}\left(x_{i}\right)$
1	$-F_{z}x_{1}-M_{y}$	$-M_{x}$
2	$-F_{z}x_{2}-M_{x}$	$F_{z}l_{1}+M_{y}$
3	$-F_{z}\left(l_{1}+x_{3}\right)-M_{y}$	$-F_{z}l_{2}-M_{x}$
4	$-F_{z}\left(-l_{2}+x_{4}\right)+M_{x}$	$-F_{z}\left(l_{1}+l_{3}\right)-M_{y}$
5	$-F_{z}\left(l_{1}+l_{3}+x_{5}\right)-M_{y}$	$F_{z}\left(l_{2}-l_{4}\right)-M_{x}$
6	$-F_{z}\left(l_{2}-l_{4}+x_{6}\right)-M_{x}$	$F_{z}\left(l_{1}+l_{3}+l_{5}\right)+M_{y}$
7	$-F_{z}\left(l_{1}+l_{3}+l_{5}+x_{7}\right)-M_{y}$	$-F_{z}\left(l_{6}-l_{4}+l_{2}\right)-M_{x}$
8	$-F_{z}\left(-l_{6}+l_{4}-l_{2}+x_{8}\right)+M_{x}$	$-F_{z}\left(l_{1}+l_{3}+l_{5}+l_{7}\right)-M_{y}$

**Table 2:** Specific strain energies $\prod_{b,i}^{*}$ and $\prod_{t,i}^{*}$ of bent and twisted beam sections *i* normalized by ${\left(2E_{\mathrm{Si}}I\right)}^{-1}$ and ${\left(2G_{\mathrm{Si}}I_{t}\right)}^{-1}$, respectively.

*i*	$2E_{\mathrm{Si}}I\prod_{b,i}$	$2G_{\mathrm{Si}}I_{t}\prod_{t,i}$
1	$l_{1}\left(l_{1}^{2}F_{z}^{2}/3+l_{1}F_{z}M_{y}+M_{y}^{2}\right)$	$l_{1}M_{x}^{2}$
2	$l_{2}\left(l_{2}^{2}F_{z}^{2}/3+l_{2}F_{z}M_{x}+M_{x}^{2}\right)$	$l_{2}\left(l_{1}^{2}F_{z}^{2}+2l_{1}F_{z}M_{y}+M_{y}^{2}\right)$
3	$l_{3}(\left(3l_{1}^{2}+3l_{1}l_{3}+l_{3}^{2}\right)F_{z}^{2}/3$ $\left(2l_{1}+l_{3}\right)F_{z}M_{y}+M_{y}^{2})$	$l_{3}\left(l_{2}^{2}F_{z}^{2}+2l_{2}F_{z}M_{x}+M_{x}^{2}\right)$
4	$l_{4}(\left(3l_{2}^{2}+3l_{2}l_{4}+l_{4}^{2}\right)F_{z}^{2}/3$ $+\left(2l_{2}+l_{4}\right)F_{z}M_{x}+M_{x}^{2})$	$l_{4}(\left(l_{1}^{2}+2l_{1}l_{3}+l_{3}^{2}\right)F_{z}^{2}$ $2\left((l_{1}+l_{3}\right)F_{z}M_{y}+M_{y}^{2})$
5	$l_{5}((3l_{1}^{2}+6l_{1}l_{3}+3l_{1}l_{5}$ $+3l_{3}^{2}+3l_{3}l_{5}+l_{5}^{2})F_{z}^{2}/3$ $+\left(2l_{1}+2l_{3}+l_{5}\right)F_{z}M_{y}+M_{y}^{2})$	$l_{5}(\left(l_{2}^{2}-2l_{2}l_{4}+l_{4}^{2}\right)F_{z}^{2}$ $2\left((l_{4}-l_{2}\right)F_{z}M_{x}+M_{x}^{2})$
6	$l_{6}((3l_{2}^{2}-6l_{2}l_{4}+3l_{4}^{2}$ $-3l_{4}l_{6}+3l_{2}l_{6}+l_{6}^{2})F_{z}^{2}/3$ $+\left(2l_{4}-2l_{2}-l_{6}\right)F_{z}M_{x}+M_{x}^{2})$	$l_{6}((l_{1}^{2}+2l_{1}l_{3}+2l_{1}l_{5}$ $+l_{3}^{2}+l_{3}l_{5}+l_{5}^{2})F_{z}^{2}$ $2\left((l_{1}+l_{3}+l_{5}\right)F_{z}M_{y}+M_{y}^{2})$
7	$l_{7}((3l_{1}^{2}+6l_{1}l_{3}+3l_{3}^{2}+6l_{1}l_{5}$ $+6l_{3}l_{5}+3l_{5}^{2}+3l_{1}l_{7}$ $+3l_{3}l_{7}+3l_{5}l_{7}+l_{7}^{2})F_{z}^{2}/3$ $+\left(2l_{1}+2l_{3}+2l_{5}+l_{6}\right)F_{z}M_{y}+M_{y}^{2})$	$l_{7}((l_{2}^{2}-2l_{2}l_{4}+2l_{2}l_{6}$ $+l_{4}^{2}-l_{4}l_{6}+l_{6}^{2})F_{z}^{2}$ $2\left(l_{2}-l_{4}+l_{6}\right)F_{z}M_{x}+M_{x}^{2})$
8	$l_{8}((3l_{4}^{2}-6l_{4}l_{6}+3l_{6}^{2}$ $+6l_{2}l_{6}-3l_{2}l_{4}+3l_{2}^{2}-3l_{6}l_{8}$ $+3l_{4}l_{8}-3l_{2}l_{8}+l_{8}^{2})F_{z}^{2}/3$ $+\left(2l_{6}-2l_{4}+2l_{2}-l_{8}\right)F_{z}M_{x}+M_{x}^{2})$	$l_{8}((l_{1}^{2}+2l_{1}l_{3}+2l_{1}l_{5}$ $+2l_{1}l_{7}+l_{3}^{2}+2l_{3}l_{5}+2l_{3}l_{7}\;$ $+l_{5}^{2}+2l_{5}l_{7}+l_{7}^{2})F_{z}^{2}$ $+\left(l_{1}+l_{3}+l_{5}+l_{7}\right)F_{z}M_{y}+M_{y}^{2})$

**Table 3:** Connection yield in % for different combinations of pad thicknesses $h_{1}$ and $h_{2}$, and beam heights $t_{b}$ for spring-suspended (bold) and stiff contact pad variants. Due to the limited magnetic force of the magnets applied, an additional load of 460 mN was exerted on top of the modules. The heights of the circular and quadratic pads on the module cover and carrier are labeled as $h_{cover}$ and $h_{carrier}$, respectively. Meander beam systems are thinned to $t_{b}$ = 200 µm except (*) with $t_{b}$ = 50 µm and (**) with $\;t_{b}$ = 100 µm.

$h_{cover\;(µm)}$	$h_{carrier\;(µm)}$			
	1	2	4	8
1	NA/7	NA/13	NA/0	NA/50
1 *	**90**/NA	**53**/NA	**40**/NA	**0**/NA
1 **	**35**/NA	**83**/NA	**100**/NA	**0**/NA
2	**94**/0	**100**/17	**94**/13	**0**/73
4	**100**/0	**100**/6	**100**/13	**0**/38
8	**84**/20	**92**/25	**92**/13	**16**/53
